# Diagnostics and Group Therapy in Patients with Persistent Postural-Perceptual Dizziness and Anxiety Disorder: Biomarkers and Neurofunctional Correlates of Underlying Treatment Effects

**DOI:** 10.3390/diagnostics15141729

**Published:** 2025-07-08

**Authors:** Maximilian Maywald, Oliver Pogarell, Agnieszka Chrobok, Susanne Levai, Daniel Keeser, Nadja Tschentscher, Boris-Stephan Rauchmann, Sophia Stöcklein, Birgit Ertl-Wagner, Boris Papazov, Marco Paolini, Susanne Karch

**Affiliations:** 1 Department of Psychiatry and Psychotherapy, LMU University Hospital Munich, 80336 Munich, Germany; 2 Department of Radiology, University Hospital, LMU University Hospital Munich, 80336 Munich, Germany

**Keywords:** persistent postural-perceptual dizziness, PPPD, functional dizziness, anxiety disorder, neuroscience, fMRI, psychotherapy, supramarginal gyrus, superior temporal gyrus, cognitive behavioural, mindfulness-based

## Abstract

**Background**: There is a certain degree of overlap between persistent postural-perceptual dizziness (PPPD) (ICD-11) and anxiety disorders (ANX) with regard to the phenomenological, pathological and neurobiological characteristics of both conditions. The implementation of an integrative psychotherapy programme may potentially result in the generation of synergistic effects across both patient groups. **Objectives**: This study assessed (1) whether psychological mechanisms similarly influence symptom severity in PPPD and ANX group, (2) the effectiveness of psychotherapy, and (3) potential neurofunctional biomarkers. **Methods**: Patients with PPPD (n = 14) and ANX (n = 20) underwent an integrative psychotherapy programme with balance training and mindfulness-based interventions. Emotional and neutral pictures were presented during MRI scans before and after therapy, with healthy controls (HC = 29) for comparison. Clinical and psychological questionnaires were administered, and brain activity was analysed in key regions. **Results**: The only diagnostic difference in the direct comparison between patients with PPPD and with ANX were the vertigo intensity values before and after therapy. PPPD with comorbid anxiety disorder had significantly more fear of physical symptoms than patients without comorbid anxiety disorder. PPPD showed no change regarding vertigo intensity (VSS), anxiety, or depression scores, but reported decreased impact of vertigo on social functioning (VHQ), and improved personal control after therapy (IPQ). By contrast, anxiety, dizziness, depression, alexithymia, and IPQ scores were significantly reduced after therapy in the ANX group. Neuroimaging revealed decreased activity in the hippocampus and superior temporal gyri (STG) in the PPPD group post-therapy as compared to the pre-therapy measurement, while the ANX group showed reduced activity in the insula, thalamus, hippocampus, and inferior frontal gyrus. Compared to the ANX and HC groups, patients with PPPD showed increased activity in the supramarginal gyrus and STG, both of which could serve as biomarkers for PPPD patients but need to be further validated. **Conclusions**: Anxiety and vertigo may reinforce each other in PPPD, as symptoms persisted post-therapy, whereas ANX patients improved significantly. Nevertheless, there is some evidence for a successful management of symptoms in the PPPD group. Findings are limited by small sample size and require further research.

## 1. Introduction

Persistent postural-perceptual dizziness (PPPD) (ICD-11) and anxiety disorders (ANX) show similarities regarding their phenomenological, pathological and neurobiological characteristics [1]. The most frequent comorbidity in PPPD is ANX, with a rate of approximately 45–60% [2,3,4]. In addition, dizziness symptoms are very common, especially in patients with agoraphobia, panic disorder, or social phobia [5,6,7]. However, there is also evidence that balance, gait, stance, and visual functions can be impaired in patients with ANX and PPPD [8,9,10,11,12,13]. Furthermore, the symptoms of anticipatory anxiety are observed in both patient groups [14,15,16]. In conclusion, patients with ANX and PPPD have multiple symptoms in common, including anxiety, avoidance, social withdrawal, hyperarousal, vertigo, palpitation, as well as stance and gait disorders.

In addition, predisposing character traits have been hypothesised to be involved in both illnesses, e.g., elevated neuroticism and introversion, and a low level of conscientiousness [17,18,19,20,21]. Additionally, elevated trait anxiety has been related to postural control disorders [20,21].

Furthermore, similarities between ANX and PPPD can not only be identified on the phenomenological level, but also in the underlying pathomechanisms. Patients with PPPD show heightened body vigilance, illness perception, or interoceptive avoidance [1,22,23,24]. An increased illness perception, or perception of physical symptoms, is also described in patients with anxiety symptoms, especially in panic disorder [1,24]. These factors are thought to lead to patients acting too early and too rigidly on their postural control after a vertigo attack. Inadequate co-contraction of antagonistic leg muscles during simple postural tasks could be found and objectified posturographically under different standing conditions [11,25,26]. All these characteristics are important in the treatment of patients with ANX and PPPD. Although there are a number of studies supporting the efficacy of selective serotonin reuptake inhibitors (SSRIs), psychotherapy, and balance training, there is a lack of treatment options for PPPD in clinical practice [27,28,29,30,31,32,33]. The efficacy of psychological therapies for ANX, e.g., psychoeducation, supportive psychotherapy, cognitive behaviour therapy (CBT), stimulation or psychodynamic therapy, was demonstrated, but effect sizes were quite heterogenous, ranging between low and high [34,35,36]. None of the mentioned studies in the meta-analysis about anxiety patients included BT, or a combination of BT and CBT, despite the fact that BT could be effective [37].

On a neurobiological basis, emotion-inducing tasks (e.g., administration of cholecystokinin-tetrapeptide (CCK-4) or presentation of emotional pictures) led to enhanced activations in anxiety networks but also in structures associated with vestibular information processing in both patient groups [1,14,38]. In a meta-analytic study, 419 anxiety patients showed decreased brain activity after psychotherapy in the insula, anterior cingulate cortex (ACC), the dorsolateral prefrontal cortex (dlPFC), and supplementary motor area. Furthermore, it has been hypothesised that a reduction in activation within the limbic (insula) and frontal brain regions (ACC) may be indicative of a therapy-related normalisation of the perception of both internal and external threats [39]. In another study hippocampus, amygdala, inferior frontal gyrus, dlPFC and ACC predicted psychotherapy outcome [40]. To date, no studies have examined the neurobiological outcome of CBT in patients with PPPD. Furthermore, there have been no studies that have directly compared anxiety patients with PPPD patients after psychotherapy. It is assumed that anxiety in particular is a key factor in the treatment and pathology of PPPD, along with other influencing factors, such as somatisation, balance deficits, visual dependency, interoceptive avoidance and illness perception.

The primary objective of the present exploratory study was to assess whether there were diagnostic differences between the HC, ANX and PPPD groups with regard to various psychological variables or symptom manifestations. It is hypothesised that the ANX group will demonstrate a higher level of anxiety in comparison to the HC and PPPD groups. Furthermore, it is hypothesised that the PPPD group will demonstrate elevated levels of dizziness and alexithymia in comparison to the HC and ANX groups. It is hypothesised that there will be no differences in depression scores or illness perception between the patient groups; however, differences are expected between the patient groups and the HC group. The second objective of the study was to evaluate the efficacy of a cognitive–behavioural psychotherapeutic programme that has been specifically designed to address both patients with anxiety disorder and PPPD, incorporating a balance training component and mindfulness-based interventions. This study was predicated on the hypothesis that both patient groups would demonstrate a reduction in symptoms (e.g., dizziness, anxiety, depression) in the pre–post comparison in the questionnaires.

The third objective of this study was to investigate the neurobiological correlates of underlying treatment effects and to find diagnostic biomarkers. It is hypothesised that alterations in neuronal activity can be measured in specific brain regions involved in emotion, visual, motor and vertigo processing. The identification of diagnostically significant biomarkers is predicted to occur in the parietal operculum and the posterior insular cortex.

## 2. Methods

### 2.1. Sample

The entirety of the data pertaining to the sample can be located in the article by Maywald et al. [1].

### 2.2. Interventions

The group therapy concept developed specifically for this study was aligned with the current state of research. It was based on existing concepts [24,30,41,42,43,44,45]. A total of 10 modular sessions at 100 min twice per week were set (Table 1). The therapy form can thus be described as high-frequency short-term therapy.

**Figure 1:**
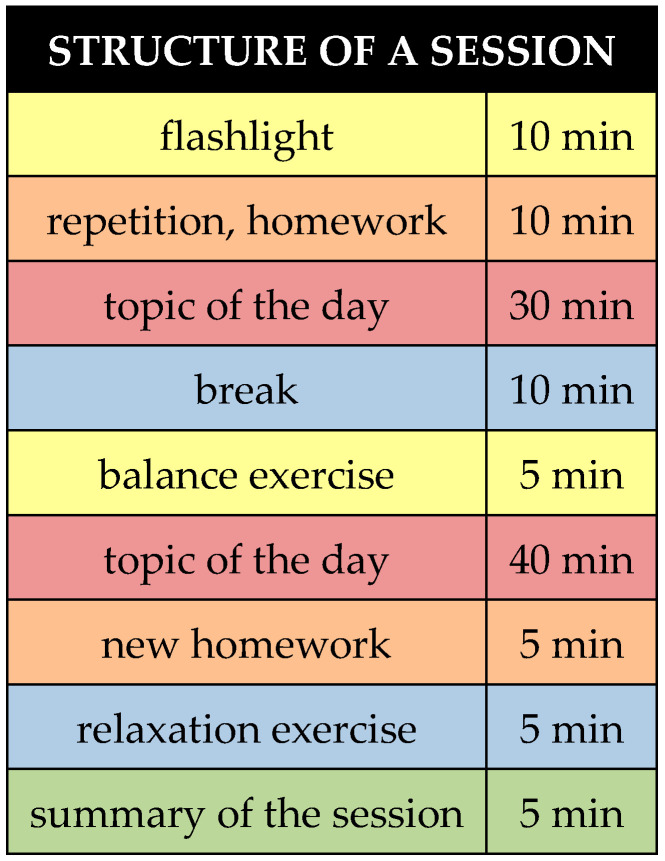
Structure of a single therapy session.

The sessions were structured and conducted according to the manual. Each session was framed by a “flashlight” in the sense of a joint opening and closing round (Figure 1). The patients were asked to reflect on what was on their mind at the time and what they had learned from the session. In addition to a daily topic, the idea was to practice at least five minutes of mindfulness or relaxation, or breathing and balance exercises in each session in the sense of regular training. For this purpose, the group received a 50-min introduction to mindfulness theory and mindfulness practice, as well as a 50-min introduction to progressive muscle relaxation [41,46]. Finally, patients were instructed to complete exercise tasks regularly and independently at home, such as keeping a dizziness or anxiety diary, as well as balance, mindfulness, and relaxation exercises. The exercise tasks were discussed in the following session in the group, and contents of the last session were repeated.

The day’s topics were worked on in partners or small groups so that as much interaction and reflection as possible could take place. This followed the paradigm of “learning through self-experience”. Content was further deepened through techniques such as guided learning, Socratic dialogue, cognitive restructuring [47], defusion techniques [48], or meta-cognitive techniques [49]. To maintain participants’ concentration for 100 min, a 10-min break was held after approximately 50 min.

### 2.3. Paradigm

For comprehensive information on MRI paradigm, please refer to the article by Maywald et al. [1].

**Figure 2:**
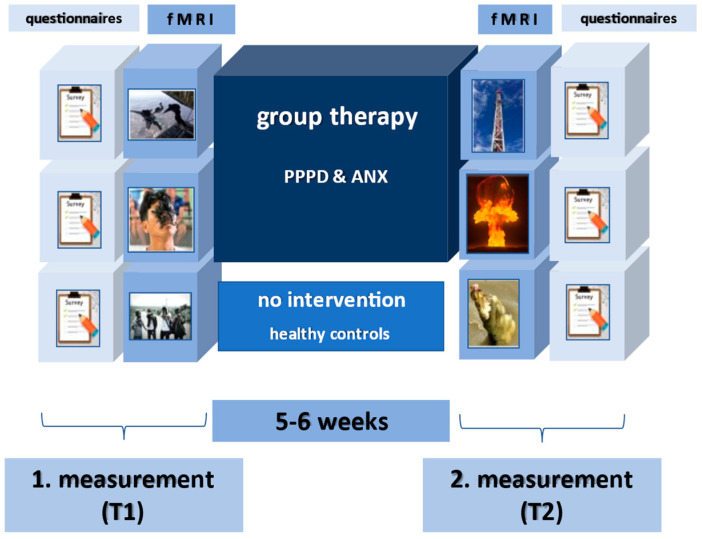
Study design: fMRI and questionnaire assessments conducted pre- and post-therapy or after a 5–6 week waiting period.

All patients underwent the fMRI paradigm before and after therapy, the healthy control group before and after a waiting period of 5–6 weeks (Figure 2).

### 2.4. Psychometric Questionnaires

A variety of psychometric tests were utilised for the purpose of evaluating a range of psychological states, including dizziness, anxiety, depression, alexithymia, and illness perception (see Table 2 for more information).

### 2.5. MRI and fMRI Data Acquisition, Pre- and Post-Processing and Statistical Analysis

For comprehensive information on MRI and fMRI data acquisition, pre- and post-processing steps, as well as the statistical analysis of functional data, please refer to the article by Maywald et al. [1]. An overview of the number of test subjects at any given time can be found in Table 3.

### 2.6. Analysis of Regions of Interest and Psychometric Data

For comprehensive information on analysis of ROI and psychometric data please refer to the article by Maywald et al. [1]. The effect sizes were calculated for the questionnaire data of the pre–post comparison exclusively for the intervention groups. They are represented by Pearson’s r, with small effect size r ≈ 0.10/−0.10, medium effect size r ≈ 0.30/−0.30 and large effect size r > 0.50/−0.50. An overview of all the regions of interest (ROI) that were calculated can be found in Table 4.

## 3. Results

### 3.1. Comparison of Psychometric Data Between PPPD, ANX and Their Particular Control Group Before and After Therapy

The GLM showed significant differences between the first and second points of measurement in the following questionnaires and its subscales: STAI-State (STAI-S), VSS ‘somatic anxiety and autonomic arousal’ (VSS-AA), VSS ‘vertigo and related symptoms’ (VSS-VER), BDI-II, IPQ ‘Personal Control’ (IPQ-PC) and ‘Coherence’ (IPQ-COH).

As demonstrated in Table 5, the post hoc tests revealed that the PPPD group exhibited significantly higher values than the HC-P group with regard to the STAI-S, STAI-T, VSS-AA, VSS-VER, VHQ, BDI-II, TAS-DIF and IPQ-TC. There was a trend-level in the IPQ-ER, but the one-way ANOVA with bootstrapping did not show any significant difference.

The post hoc tests indicated that the ANX group exhibited significantly higher questionnaire scores compared to the HC-A group in the STAI-S, STAI-T, VSS-AA, BDI-II, TAS total, TAS-DIF, TAS-DDF, IPQ-TL a/c, and IPQ-ER (Table 6). There was a trend-level in the VSS-VER, but the one-way ANOVA with bootstrapping did not show any significant difference.

Post hoc tests did not show any difference between the PPPD and the ANX group, but there was a trend-level in the VSS-VER (Table 7). After conducting a one-way independent ANOVA with bootstrapping, there was a significant difference between the PPPD and the ANX group in the VSS-VER subscale (Table 8). Furthermore, in the BSQ there was a significant difference between the PPPD groups with and without comorbid anxiety disorder (Table 9).

### 3.2. Questionnaires in a Before-and-After Comparison

The before–after comparison for individual groups showed no significant differences in HC-P, except in HC-A in BDI-II. However, the values were in the non-clinical range. The PPPD group showed a significant increase in IPQ personal control scores, and a significant reduction in VHQ scores. The anxiety group showed significantly reduced BDI-II, STAI-S, VSS-AA, VSS-VER, and IPQ emotional representation scores, and significantly increased IPQ personal control, cure control, and coherence scores. In the anxiety group, effect sizes were in the small-to-medium range in the anxiety questionnaires (STAI-S and STAI-T), in the small-to-high range in IPQ Timeline, Time Cyclical, Consequence, Personal Control, Cure Control, Coherence, Emotional Representation, and in the small-to-medium range in the dizziness questionnaires (VHQ, VSS-AA, VSS-VER). In the dizziness group, effect sizes were in the small-to-medium range in anxiety, dizziness, and illness perception questionnaires (Table 10).

### 3.3. ROI Analysis Before and After Therapy

The comparison of ROI data revealed significantly reduced neuronal activity in the hippocampus (*p*-value = 0.02), thalamus (*p*-value = 0.01), inferior frontal gyrus (IFG, *p*-value = 0.03), and insula (*p*-value = 0.04) in the second as compared to the first measurement in the anxiety disorder group (Wilcoxon test for connected samples, Table 11). In addition, after conducting an ANOVA with bootstrapping, the activity in the amygdala was also significantly reduced (Table 12). Differences in the PPPD group and healthy control group were not significant (Table 11), but after conducting an ANOVA with bootstrapping, the activity in the hippocampus was also significantly reduced in the PPPD group (Table 13).

### 3.4. Comparison of Neuronal Responses Before and After Therapy—PPPD

**Figure 3:**
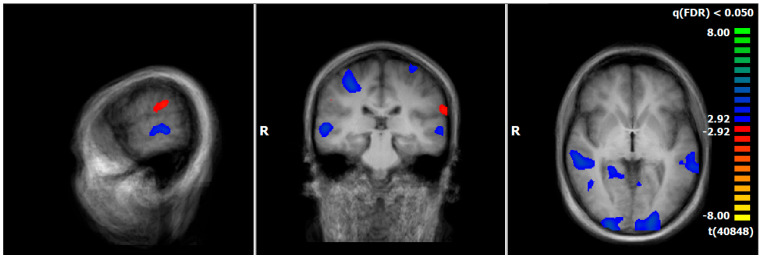
PPPD-T1 vs. T2, neural responses of the emotion-associated [negative emotional pictures > neutral pictures; q(FDR) < 0.05, T-score: −8 to 8, fixed-effects-analysis], in red: increase in neuronal response, e.g., supramarginal gyrus; in blue: decrease of neuronal activations, e.g., parahippocampal gyrus, superior/middle temporal gyrus (x = −60; y = −26; z = 0).

Comparing pre–post measurements, PPPD showed a decrease in neural responses after therapy, especially in brain regions associated with the processing of vestibular (superior temporal gyrus [STG, BA22], cuneus), emotional (hippocampus, parahippocampal gyrus), visual (occipital gyrus), and motoric information (precentral gyrus, cerebellum) during the emotion-associated task (negative emotional pictures minus neutral pictures). There was an increase in neuronal response in brain structures related to processing of vestibular information (supramarginal gyrus [SMG, BA40]) (Figure 3, Table S1).

### 3.5. Comparison of Neuronal Responses Before and After Therapy—ANX

**Figure 4:**
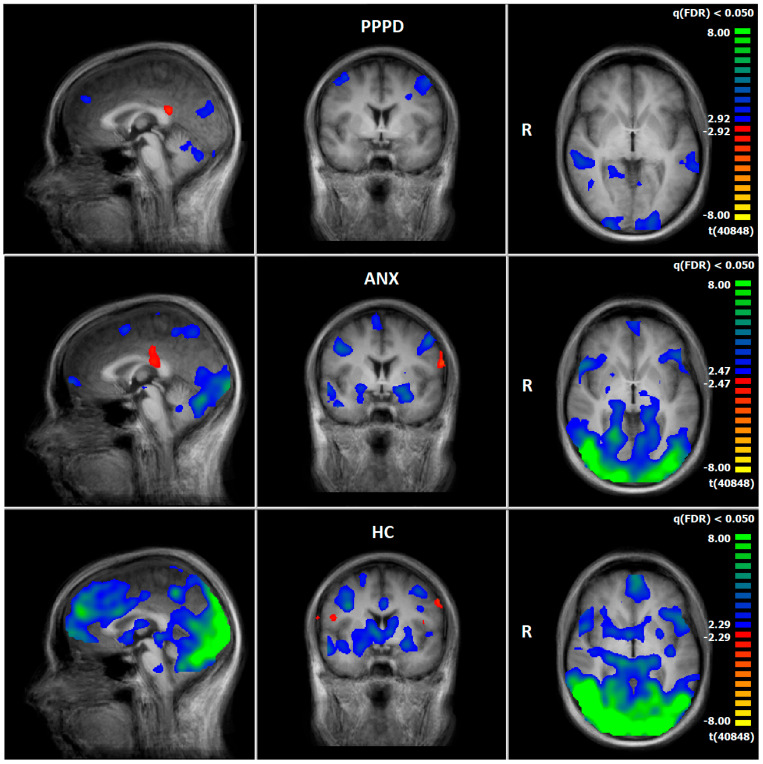
T1 vs. T2, neural responses of the emotion-associated [negative emotional pictures > neutral pictures; q(FDR) < 0.05, T-score: −8 to 8, fixed-effects-analysis], in red: increase of neuronal response; in blue–green: decrease of neuronal activations (x = 0; y = 0; z = 0). First row: PPPD group; second row: ANX group; third row: HC group.

Comparing pre–post measurements, ANX showed a decrease in neural responses after therapy, especially in brain regions associated with the processing of vestibular (precuneus, STG), emotional (amygdala, insula, parahippocampal gyrus, lentiform gyrus), visual (Middle Occipital Gyrus), and motoric information (precentral gyrus, cerebellum) during the emotion-associated task (negative emotional pictures minus neutral pictures). There was an increase in brain structures related to higher cognitive control functions, such as the dlPFC (BA9/10) (Figure 4, Table S2).

### 3.6. HC: Pre–Post Comparison of Neuronal Responses Following a 5–6-Week Interval

Comparing pre–post measurements, HC showed a decrease in neuronal response at T2, especially in brain regions associated with the processing of vestibular (precuneus, cuneus, STG, SMG), emotional (amygdala, insula, parahippocampal gyrus, lentiform gyrus), visual (occipital gyrus), and motoric information (precentral gyrus, cerebellum) during the emotion-associated task (negative emotional pictures minus neutral pictures). There was an increase in brain structures related to processing emotional information, such as the insula or IFG (Figure 4, Table S3).

### 3.7. Post-Treatment Comparison of Neuronal Responses Between PPPD and ANX

**Figure 5:**
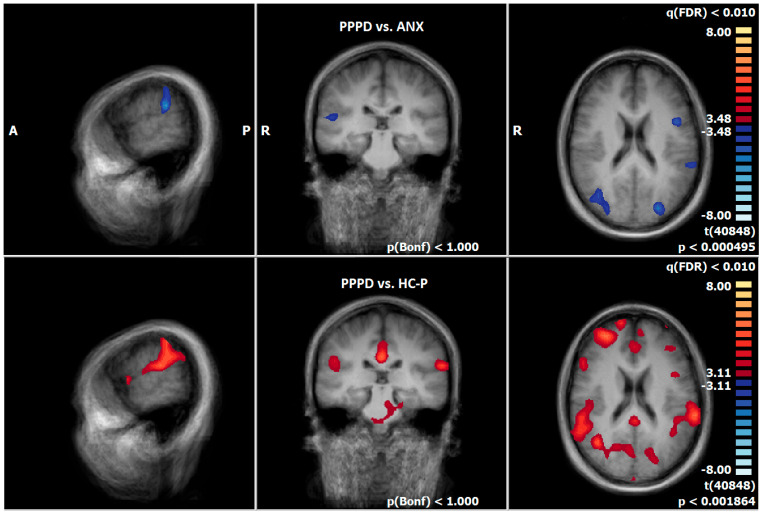
First row: T2-PPPD vs. ANX group; second row: T2-PPPD vs. HC-P group; neural responses of the emotion-associated [negative emotional pictures > neutral pictures; q(FDR) < 0.01, T-score: −8 to 8, fixed-effects-analysis], in blue: PPPD > ANX group e.g., left supramarginal gyrus; in red: PPPD > HC-P group, e. g. left and right supramarginal gyrus (x = −60; y = −26; z = 20).

After therapy, PPPD showed an increased neuronal response, especially in brain regions which are associated with the processing of vestibular (SMG, STG, precuneus, cuneus,), emotional (parahippocampal gyrus, IFG), visual (fusiform gyrus), and motoric information (precentral gyrus, cerebellum), compared to ANX during the emotion-associated task (negative emotional pictures minus neutral pictures) (Figure 5, Table S4). In contrast, there was no increased neuronal response in ANX compared to PPPD.

### 3.8. Post-Treatment Comparison of Neuronal Responses Between PPPD and HC-P

**Figure 6:**
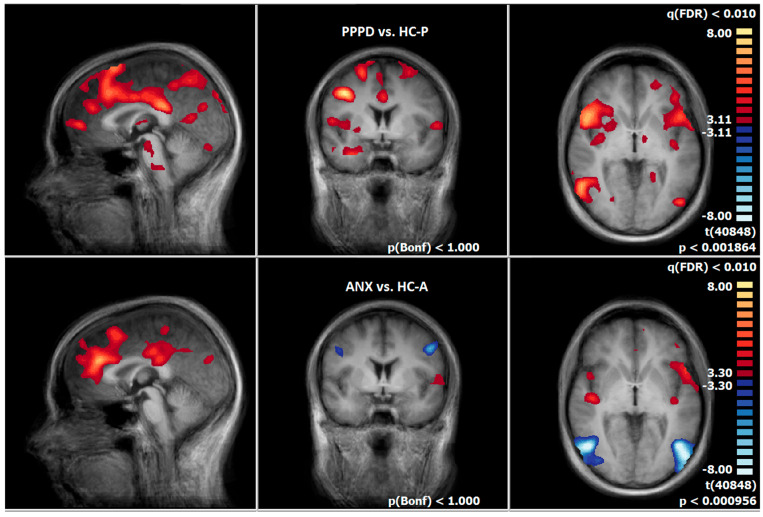
First row: T2-PPPD vs. HC-P group; second row: T2-ANX vs. HC-A group, neural responses of the emotion-associated [negative emotional pictures > neutral pictures; q(FDR) < 0.01, T-score: −8 to 8, fixed-effects-analysis], in red: increased neuronal response of the patient groups, e.g., anterior and posterior insula (x = 0; y = 0; z = 0).

After therapy, PPPD showed an increased neuronal response compared to HC-P, particularly in brain regions associated with the processing of vestibular (SMG, precuneus), emotional (insula, amygdala, lentiform gyrus, parahippocampal gyrus, IFG), visual (fusiform gyrus), and motoric information (precentral gyrus, cerebellum) during the emotion-associated task (negative emotional pictures minus neutral pictures, Figure 6, Table S5).

### 3.9. Post-Treatment Comparison of Neuronal Responses Between ANX and HC-A

After therapy, ANX showed an increase in neuronal response compared with HC-A, particularly in brain regions associated with the processing of emotion (left posterior insula, cingulate gyrus, IFG) during the emotion-associated task (negative emotional pictures minus neutral pictures). HC-A showed stronger neuronal activation than ANX in visual processing brain regions (occipital gyrus) (Figure 6, Table S6).

## 4. Discussion

To our knowledge, this is the first study comparing directly the neurobiological basis of emotion processing in patients with persistent postural-perceptual dizziness (PPPD) and anxiety disorders (ANX) following integrative cognitive behavioural group therapy, focusing on brain regions involved in emotional, vestibular, and visual networks.

### 4.1. Clinical Findings from Psychometric Analysis

Both PPPD and ANX patients showed significantly higher state and trait anxiety scores (STAI) than their respective control groups, but there was no significant difference between the two patient groups. This indicates elevated anxiety as both a state and trait in both conditions, which is consistent with previous research [14,21,65]. The assumption that individuals diagnosed with anxiety would score higher in anxiety traits or state scores than those with PPPD had to be rejected. Some studies suggest the scale may reflect general neuroticism rather than anxiety alone [66,67]. Higher neuroticism in patients with chronic subjective dizziness was reported, for example, by Chiarella, Petrolo, Riccelli, Giofrè, Olivadese, Gioacchini, Scarpa, Cassandro and Passamonti [20], or in PPPD by [22].

After therapy, anxiety scores (STAI-S) significantly decreased in the ANX group but not in the PPPD group, suggesting greater therapeutic benefit for the ANX group. Similar findings have been reported in other studies using comparable therapies [28,45].

Both patient groups had higher Anxiety Cognition Questionnaire scores than controls, indicating comparable levels of anxious thoughts, but did not differ from each other. PPPD scores matched those of the norm sample seen in “other anxiety disorders”, resembling phobias. Only the ANX group showed a reduction in ACQ scores after therapy, suggesting greater treatment effectiveness for ANX.

Both patient groups scored higher than controls on the Body Sensation Questionnaire, with no significant difference between them. PPPD patients with comorbid anxiety showed even greater anxiety about bodily sensations, matching reference values for panic/agoraphobia, while those without comorbid anxiety matched values for “other anxiety disorders”. This highlights the overlap between PPPD and anxiety disorders, particularly with regard to concerns about physical symptoms in patients with a comorbid anxiety disorder.

The Mobility Inventory A & B (avoidance behaviour alone or accompanied) showed no significant difference between groups (PPPD, HC, anxiety). We suggest that new diagnostic questionnaires are needed to measure avoidance behaviour in patients with PPPD.In summary, the anxiety group showed lower anxiety scores (STAI-S, ACQ, BSQ) after therapy as compared to before, while the results of the PPPD patients did not change. This may indicate a greater reduction in symptoms in ANX than in PPPD. The lack of improvement in PPPD, with or without possible comorbid anxiety, suggests that anticipatory anxiety or comorbid anxiety may contribute to the maintenance of PPPD symptoms and limit treatment effects. The second hypothesis, that psychotherapy is similarly effective, had to be rejected. Moreover, the first hypothesis that individuals diagnosed with anxiety score higher in anxiety traits or state scores than those with PPPD had to be rejected.

The subscale somatic anxiety and autonomic arousal of the Vertigo Symptom Scale revealed higher physical anxiety symptoms in both patient groups as compared to HC-A and HC-P after therapy, though only the anxiety group showed a significant reduction. Regarding vertigo symptoms, the ANX group no longer differed from HC-A post-therapy, while PPPD patients still had elevated scores compared to HC-P and to the ANX group. The hypothesis, which postulated that the PPPD group would demonstrate a higher prevalence of dizziness symptoms, can be maintained.

The pre–post comparisons demonstrated that the dizziness symptomatology was significantly reduced after therapy in the ANX group. The ANX group showed a medium effect size, while the PPPD group showed a low-to-medium effect size. This is congruent with other studies [28,45]. Limburg et al. [28] could not measure a significant decrease directly after the end of therapy, but a significant decrease was shown catamnestically after 12 months. Other studies showed that psychotherapeutic interventions can have an effect after 6–12 months [68,69]. Whether psychotherapeutic effects also occurred in the current study after 6–12 months can only be speculated due to the lack of a catamnesis.

Regarding the Vertigo Handicap Questionnaire, the dizziness group demonstrated a significant reduction in vertigo-related handicap after therapy, exhibiting a medium effect size. This improvement was primarily characterised by an enhancement in their capacity to manage the social impact of vertigo. This finding stands in contrast to the results reported in the study conducted by Tschan, Eckhardt-Henn, Scheurich, Best, Dieterich and Beutel [45]. This suggests that, while symptom intensity did not decrease, PPPD patients gained better coping and self-regulation skills, supporting findings from Limburg et al. [28].

In the Beck Depression Inventory II, the anxiety and dizziness groups showed clinically relevant scores that were significantly higher than those of the control groups [1]. Pre–post comparisons showed a significant decrease in the ANX group. In the study by Limburg, Radziej, Sattel, Henningsen, Dieterich, Probst, Dale and Lahmann [28], the dizziness group also did not show a significant decrease in symptoms at the second survey time point, but did at the 12-month follow-up.

In the Toronto Alexithymia Scale the ANX group had higher alexithymia scores than controls (total scale TAS and subscales DIF and DDF), while PPPD differed from HC-P in difficulty recognizing feelings. Despite previous findings that alexithymia predicts poorer therapy response [70], the ANX group—though more alexithymic—responded better to therapy. From a psychoanalytic perspective, the construct is more related to the psychological defence mechanism of “affect isolation” (separating emotions from thoughts) than to the defence mechanism of “somatisation”, in which a conflict is displaced into the body. Thus, if alexithymia is considered an adaptation to difficult developmental conditions [71,72], it is not per se something specific to functional disorders, but to psychiatric and psychosomatic disorders alike.

In the Perception Illness Questionnaire, both the anxiety and dizziness groups demonstrated a significant improvement on the subscale “Personal Control” following therapy, indicating an increased sense of influence over symptoms. The magnitude of this effect was large in the anxiety group and medium in the dizziness group, aligning with the findings of previous research [45]. In addition, the anxiety group improved their understanding of the disease (coherence), the emotional handling of the symptoms—e.g., “disease makes angry”, “afraid” or depressed” (emotional representation)—as well as gained more confidence in the treatment of the disease (treatment control). While both groups benefited, the anxiety group showed greater reductions in symptoms and better emotional understanding. The hypothesis that both groups would benefit equally from therapy is only partially supported.

### 4.2. Functional Imaging Data

#### 4.2.1. Emotion-Processing Network

Patients and HC demonstrated decreased neuronal responses in T2 compared to T1 in brain areas related to emotional networks. Surprisingly, the HC group (HC-A + HC-S) showed the most significant decrease, e.g., in the amygdala, globus pallidus, hippocampus, dlPFC (BA8, BA9), IFG (BA47, BA45), ACC (BA32), insula (BA13), caudate nucleus, superior temporal gyrus (BA22, BA38), pons, and thalamus. A possible explanation could be that healthy individuals adapted better to emotionally challenging images than patients. Habituation to emotionally relevant information and reduced neurobiological responses in healthy controls have been previously reported [73,74,75]. Patients in the ANX group demonstrated decreased responses, e.g., in the amygdala, globus pallidus, hippocampus, IFG (BA47, BA45), STG (BA38), OFC (BA10), insula (BA13), nucleus caudatus, and thalamus, the PPPD group in the dlPFC (BA9), superior temporal gyrus (BA 22), hippocampus, and parahippocampal gyrus. These brain regions correspond to the anxiety networks found in previous studies [76,77,78,79,80]. However, these brain regions partly overlap, e.g., with the vestibular network. The direct comparisons between the HC group and the patients demonstrated stronger neuronal responses in patients in the insula and the IFG at T2 (the results of T1 can be found in Maywald et al. [1]). These results are in line with those of the questionnaires: patients of the ANX group reached increased anxiety scores compared to the standard values at T2. Following therapy, the ANX group displayed diminished neuronal reactions in emotional networks, compared to the PPPD group. However, no such observations were witnessed at T1 [1]. Overall, the results suggest that the therapeutic programme seemed to be more effective in patients with an anxiety disorder than in patients with PPPD. These findings were supported by ROI analyses, which demonstrated a significant decrease in the insula, hippocampus, thalamus, IFG, cingulate gyrus, and amygdala in the ANX group. Comparable results are also presented in the literature [76,78,81,82]. The PPPD group showed more increased neuronal activity in brain structures that are related to emotional processes, e.g., the parahippocampal gyrus/amygdala than ANX group at T2. Consequently, the hypothesis, that CBT is equally effective in patients with ANX as in patients with PPPD, has to be rejected.

Reduced emotional processing in PPPD may be linked to decreased activation in the hippocampus and parahippocampal gyrus. A meta-analysis showed increased amygdala and hippocampal/parahippocampal responses to unpleasant stimuli across several psychiatric disorders [83]. We observed a similar increase in PPPD patients at baseline [1]. However, given our emotional-image paradigm, we suggest that reduced activity in these regions reflects a diminished emotional response in PPPD, although PPPD patients still showed greater activation than HC and ANX groups at T2. Additional validation is required to determine the extent to which this effect is clinically relevant.

An increased neural response in the right posterior cingulate cortex (PCC) was observed in the PPPD and ANX groups after therapy. In contrast, the HC group did not show comparable variations at T2, and there was no clear difference at T1 [1]. The PCC plays a central role in the default mode network and is part of the fronto-parietal control network [84,85]. There is no common consensus about its functions and role in psychiatric illness, but it is involved, for example, in anxiety, schizophrenia, depression, Alzheimer’s disease and attention deficit hyperactivity disorder. Increased PPC activity is reported in a fear conditioning task with non-painful stimuli [86]. Other authors report changed PCC activity after therapy, and interpret their results as newly learned reappraisal strategies [87]. There are various explanations for the differences between the groups, e.g., the neural activity of the PCC could reflect a learning process. Otherwise, it is possible that emotional stimuli may hold greater emotional valence for the patient group than for the HC group, which was masked by a primacy effect at T1 in the HC group. This assumption is reinforced by the involvement of the PCC and the amygdala in the neural processing of first impressions [88].

In terms of a therapy effect, the anxiety group showed increased neuronal responses in frontal regions (medius frontal gyrus [BA8/dlPFC bilateral], and superior frontal gyrus [BA9/dlPFC left and BA10/oPFC right]) at the pre–post comparison, which is consistent with other therapy studies [82,89]. This finding could be interpreted as increased cognitive control over emotions or anxiety extinction. Furthermore, this finding is consistent with the questionnaire data of this study, which showed a symptom reduction, better coping strategies and better understanding of emotions.

#### 4.2.2. Vestibulo-Spatial Network

The right SMG (BA40) displayed increased activity in PPPD at T1 [1] and T2, compared to ANX and the healthy control group. This finding is consistent with the conclusions of earlier studies that identified the right supramarginal gyrus (SMG) as a pivotal region for the processing of vestibular information, with an integrative function of vision, proprioception, and movement, and as part of the retroinsular cortex [62,90,91,92,93]. In this sense, the third hypothesis was confirmed, namely that neuronal activation in the SMG, as part of the retroinsular cortex, is more pronounced in the PPPD group than in the HC and ANX groups. In our previous study, it was assumed that the PPPD group may be more sensitive to the visual–spatial and movement information of individuals depicted in pictures, which could potentially elicit their own vestibular symptoms, such as those induced by roller coaster rides [1]. On the other hand, it is possible that this trend may have even intensified during the 5–6-week period, especially in isolated individuals who are considered outliers. While the average scores in the questionnaires did show slight signs of decrease, these were not statistically significant.

ANX showed decreased neuronal activity in the retroinsular cortex in the pre–post measurement, which could be a neural correlate that the anxiety subjects reduced their dizziness symptoms, which is in accordance with the literature [8,9] and the questionnaire data of the current study.

The superior temporal gyrus is associated with the nonspecific vestibulo-spatial network [1,90,94] and is located next to the SMG [64,95,96]. It is described as a multisensoric hub [97]. The STG is also related to the anxiety network, because of its strong functional connectivity to the amygdala [79]. There is evidence for its involvement in patients with vertigo [98,99], in movement perception (BA22) [100], and the recognition of emotional expression of faces and emotional processing (BA38) [101,102]. In the pre–post comparison, the patients with PPPD showed significantly reduced responses in this area. These results may indicate a less emotional involvement in interpreting vestibular information. Furthermore, the PPPD group showed a stronger activation in the STG in comparison to ANX and HC, which can be interpreted as an increased neural response to vestibular and emotional stimuli in the PPPD group. Corresponding results were already apparent before the therapy [1]. HC and ANX groups showed a decreased BOLD signal in this area after the treatment, and even in the direct comparison there was no difference anymore, thus we suppose that this is a brain region involved in the general processing of emotional and vestibular information and that HC and ANX groups adapted better than the PPPD group.

While at T1 there was no clear difference in the precuneus between the groups [1], this changed at T2. Patients with PPPD revealed stronger neuronal activity in the left (BA7) and right (BA7 and BA31) precuneus in comparison to ANX and HC-P. These changes could be influenced by habituation. It has been shown that electrical stimulation of the precuneus can lead to vestibular symptoms, suggesting that the precuneus is involved in processing vestibular information [95]. Li et al. [103] showed reduced low-frequency fluctuation and regional homogeneity in the right precuneus in patients with PPPD compared to controls during resting state. In another study Li et al. [104] identified an altered connectivity between the precuneus and the posterior default mode network, bilateral precuneus, and premotor cortex. These neural changes were interpreted as correlates of abnormal postural control and impaired integration of internal and external spatial information in PPPD. In addition, in visual and motor integration tasks, the precuneus is also thought to be involved in reflective self-awareness [105] and emotional attribution of one’s emotional state [106]. We hypothesise that PPPD patients are more attentive and sensitive to stressful bodily sensations and visual stimuli which can disrupt balance and trigger vestibular symptoms, and that this is reflected, among other things, in increased precuneus activity. This would be congruent with the concept of somatosensory amplification and why distraction tasks improve balance function in PPPD [107,108,109].

#### 4.2.3. Visual Network

The neuronal activity in the visual structures, e.g., the fusiform gyrus and the occipital regions, was more pronounced in the HC group than in the patient groups at T1 [1] and T2. We assumed that this may indicate a better adaption to emotional stimuli, which could be shown also in other studies [73,74,75]. However, at T2 the PPPD group showed stronger activations in the visual processing areas than the ANX group, which could be a hint for visual dependency. This was not explicitly measured, but other authors report findings in patients with dizziness which show alterations in the visual system [92,110,111,112].

## 5. Limitations

This exploratory study is limited by small group sizes, reduced statistical power and potentially affected effect sizes. To address this, we focused on consistency between our statistical and neurobiological findings, compared our results with previous studies, and used statistical bootstrapping to increase power. Furthermore, no randomisation or blinding was possible, which carries the risk of overestimating the measured effects [113]. In addition, no covariates were added to the calculation, as anxiety and depression may be confounded with PPPD, i.e., people suffering from PPPD are more likely to develop depressive and anxiety symptoms [2]. The images were not subdivided into general anxiety-inducing and dizziness-specific categories, which may have limited insights into neurofunctional processing. All groups showed decreased neuronal activity from T1 to T2. Since HCs received no treatment, this suggests confounding factors, such as methodological artifacts or habituation effects. To address this, different image sets were used at each time point. Given the study design, sequence effects should be consistent across groups. Thus, if therapy is equally effective for ANX and PPPD, changes in neuronal activity should be similar in magnitude but may differ by brain region.

## 6. Conclusions

It is hypothesised that anxiety and vertigo symptoms have a negative reinforcing effect on each other in people diagnosed with PPPD, because symptoms have not decreased after therapy, like in the ANX group. It is imperative that therapists direct their attention towards subgroups of diagnostic interest, such as comorbid anxiety disorder, when formulating treatment plans. This is particularly salient in cases where symptoms such as fear of bodily sensations and avoidance behaviour are present, as these have been shown to impede the process of remission. The SMG and STG demonstrated the most substantial disparities in neuronal activity following treatment within the PPPD group when compared to the ANX and HC groups. This finding suggests that these two structures may be of particular importance as a biomarker for diagnostics and treatment outcome; these findings need to be further validated. A decrease in neuronal activity in the STG, hippocampus, and parahippocampal gyrus following pre- and post-therapy comparisons might also serve as a neuronal marker for the effectiveness of the treatment and needs further investigation. Only the anxiety group managed to significantly reduce symptoms after psychotherapy. Nevertheless, the PPPD group also showed positive tendencies in the management of symptoms that may indicate a therapeutic effect.

Hence, it is recommended that future studies incorporate a more protracted intervention period, a catamnesis phase, augmented cohorts, a study design controlling for confounding variables and novel questionnaires such as the NPQ. Furthermore, a combination of treatment modalities is advocated, including psychotherapy, SSRIs, tDCS and balance training, as Suica, Behrendt, Ziller, Gäumann, Schädler, Hilfiker, Parmar, Gerth, Bonati and Schuster-Amft [36] have proposed.

## Figures and Tables

**Table 1:** Summary of Therapy Session Content.

1. session	**Topic**: *Introduction to the topic of anxiety and vertigo, and mindfulness* Symptoms, origins, causes, principles of mindfulness theory and exercises.
2. session	**Topic**: *Explanatory models on the topic of anxiety and dizziness* When anxiety/dizziness is useful/becomes a problem, vulnerability stress model, bio-psycho-social conditioning, bio-psycho-social disease model, vicious circle of anxiety/dizziness, primary/secondary illness gain.
3. session	**Topic**: *Strategies to deal with thoughts* Recognizing anxiety thoughts, changing anxiety thoughts, PMR.
4. session	**Topic**: *Strategies for dealing with emotions* Recognizing and understanding emotions, emotion management, mindfulness-based acceptance and commitment strategies.
5. session	**Topic**: *Exposure with reaction prevention* Recognizing and understanding avoidance behaviours, anxiety hierarchy, anticipatory anxiety, provocation exercises (in sensu, in vivo, in a group and individual setting).
6–8. session	**Topic***: individual therapy in a group setting* Individual history of symptom development and deriving new strategies.
9. session	**Topic**: *resources* Activation of strengths, three pillars model of self-esteem, reframing techniques, resource exercises, gratitude diary.
10. session	**Topic**: *dealing with setbacks* Relapse prevention, emergency kit, early warning symptoms, saying goodbye.

**Table 2:** Questionnaires.

Questionnaire	Scale	Abbreviation
State Trait Anxiety Inventory [50] Subscales: Trait (anxiety as character trait, stable over time) State (anxiety as a mental state)	1 (not at all) to 4 (very much)	STAI STAI-T STAI_S
Fragebogen zu körperbezogenen Ängsten, Kognitionen & Vermeidung [51] Subscales: Agoraphobic Cognitions Questionnaire (e.g., catastrophic thinking) Body Sensations Questionnaire (fear of body symptoms) Mobility Inventory (avoidance behaviour)	1 (not at all) to 5 (extreme)	AKV ACQ BSQ MI
Vertigo Handicap Questionnaire [52]—Physical and psychosocial impairment due to dizziness	0 (never) to 4 (always)	VHQ
Vertigo Symptom Scale [53] Subscales: Somatic anxiety and autonomic arousal (measures physical anxiety symptoms) Vertigo and related symptoms (measures dizziness severity)	0 (never) to 4 (very often)	VSS VSS-AA VSS-VER
Beck Depressions Inventory II [54]	0 to 4	BDI-II
Toronto Alexithymia Scale Subscales: Difficulty identifying feelings Difficulty describing feelings	1 (strongly disagree) to 5 (strongly agree)	TAS TAS-DIF TAS-DDF
Illness Perception Questionnaire [55] Subscales: Timeline acute/chronic (belief if illness last short or long time) Timeline cyclical (symptoms come and go) Consequences (how illness effects life) Personal control (to improve symptoms) Cure control (e.g., treatment will be effective) Coherence (e.g., illness is a mystery) Emotional representation (how illness effects emotions)	1 (not true at all) to 5 (completely true)	IPQ IPQ-T a/c IPQ-TC IPQ-CON IPQ-PC IPQ-CC IPQ-COH IPQ-ER

**Table 3:** Number of MRI and questionnaire datasets at different measurement time points.

Patients	fMRI-Pre	fMRI-Post	Q-Pre	Q-Post
PPPD	10	11	14	11
ANX	13	11	20	16
total	24	22	34	27
**Healthy Controls**				
HC-P	9	8	13	10
HC-A	15	15	16	16
Total	24	23	29	26

Abbreviations: MRI: functional magnetic resonance imaging, Q: questionnaires, pre: first time point of measurement before therapy, post: second time point of measurement after therapy.

**Table 4:** Presents the relevant ROIs utilised in the study.

ROI	Abbreviation	Relevance
Amygdala	AMY	Emotion processing, threat detection, emotional memory, fear learning and conditioning [56]
Cingulate gyrus	CG	Emotion processing/regulation, behavioural regulation, learning, cognitive processing [57]
Hippocampus	HIP	Emotion memory, new memories (declarative and episodic), spatial navigation [58]
Inferior frontal gyrus	IFG	Language, inhibition and cognitive control, social cognition [59,60], decreased after CBT in patients with AD [40]
Insula	INS	Interoception, emotion processing/regulation, autonomic regulation, cognitive control, taste and motivation [61]
Supramarginal gyrus	SMG	Integrates multiple sensory modalities, including proprioceptive (body position), auditory, visual, vestibular and somatosensory information, empathy and social cognition, phonological processing [61,62,63,64]

**Table 5:** Post hoc test of psychometric data between HC-P and PPPD.

Questionnaire	HC-P				PPPD				*p*-Value
	T1		T2		T1		T2		
	M	SD	M	SD	M	SD	M	SD	
STAI-S	34.40	9.79	33.10	9.11	47.55	11.29	45.27	8.51	<0.01 *
STAI-T	45.70	3.53	46.10	2.13	49.00	3.13	49.91	1.45	0.02 *
ACQ	1.26	0.25	1.15	0.15	1.74	0.51	1.44	0.24	0.04 *
BSQ	1.71	0.71	1.29	0.31	2.38	0.70	1.98	0.53	0.01 *
MI-A	1.32	0.33	1.16	0.23	1.69	0.78	1.36	0.42	0.25
M-B	1.04	0.12	1.02	0.03	1.46	0.63	1.19	0.28	0.10
VSS-AA	7.40	5.17	4.60	3.92	25.91	10.52	24.64	10.92	<0.01 *
VSS-VER	2.40	2.12	1.00	0.82	27.64	17.40	25.73	18.80	<0.01 *
VHQ	11.50	14.58	10.70	12.53	43.45	20.05	34.82	19.98	<0.01 *
BDI-II	3.10	3.63	2.80	3.49	14.00	7.18	11.09	5.38	<0.01 *
TAS-total	53.40	8.36	52.90	5.49	59.82	9.14	59.27	9.81	0.49
TAS-DIF	13.60	4.14	13.50	2.88	20.55	5.92	20.00	6.80	0.02 *
TAS-DDF	12.20	3.16	11.20	3.33	13.09	3.75	13.00	4.29	1.00
IPQ-TL a/c	13.00	3.74	12.10	4.07	16.73	3.95	14.73	2.90	0.16
IPQ-TL c	9.90	2.60	9.70	3.40	13.00	2.72	12.64	2.25	0.04 *
IPQ-CON	12.30	4.27	12.60	4.30	16.73	2.97	15.09	3.59	0.17
IPQ-PC	13.20	1.81	13.30	2.67	12.82	2.71	15.18	4.29	1.00
IPQ-CC	13.70	2.83	14.40	2.91	13.73	1.74	14.45	3.91	1.00
IPQ-COH	16.50	2.99	18.20	3.91	15.18	5.31	16.55	4.87	1.00
IPQ-ER	12.20	4.02	13.20	4.44	18.18	5.72	16.36	4.43	0.06

Abbreviations: * significant, IPQ-DIF: difficulty identifying feelings, DDF: difficulty describing feelings, TL a/c: timeline acute/chronic, TL c: timeline cyclical, CON: consequences, PC: personal control, CC: cure control, COH: coherence, ER: emotional representation.

**Table 6:** Post hoc test of psychometric data between HC-A and ANX.

Questionnaire	HC-A				ANX				*p*-Value
	T1		T2		T1		T2		
	M	SD	M	SD	M	SD	M	SD	
STAI-S	33.38	8.56	29.31	7.29	47.06	11.20	37.94	10.21	<0.01 *
STAI-T	46.81	3.35	45.56	3.63	50.19	3.90	49.75	2.79	<0.01 *
ACQ	1.27	0.32	1.12	0.16	1.92	0.72	1.56	0.41	0.02 *
BSQ	1.66	0.64	1.28	0.27	2.36	0.84	1.80	0.68	0.01 *
MI-A	1.44	0.60	1.26	0.43	2.31	0.96	1.87	1.00	0.03 *
MI-B	1.09	0.11	1.05	0.10	1.90	0.73	1.38	0.56	0.01 *
VSS-AA	8.31	9.96	8.31	9.70	24.06	11.42	18.19	6.78	<0.01 *
VSS-VER	3.88	4.46	3.38	3.74	13.00	12.16	7.75	8.33	0.08
BDI-II	3.25	3.89	1.88	3.56	13.13	10.31	8.00	7.46	<0.01 *
TAS-total	50.88	9.38	50.88	9.22	63.25	9.67	62.25	7.65	<0.01 *
TAS-DIF	14.13	4.10	13.38	5.23	21.63	6.62	20.25	5.29	<0.01 *
TAS-DDF	10.50	3.386	10.56	3.52	15.00	3.93	15.12	4.00	<0.01 *
IPQ-TL a/c	12.62	5.23	12.69	4.63	16.25	2.41	15.44	3.98	0.04 *
IPQ-TL c	10.62	2.99	10.31	3.18	11.94	2.67	11.81	2.14	0.65
IPQ-CON	12.31	4.24	12.75	4.93	16.87	3.40	14.63	4.90	0.07
IPQ-PC	14.50	3.74	14.44	3.12	12.69	2.85	15.50	1.97	1.00
IPQ-CC	14.06	3.86	14.19	3.21	13.69	1.70	15.00	1.79	1.00
IPQ-COH	17.06	4.95	17.37	3.93	13.69	3.91	17.94	3.97	1.00
IPQ-ER	12.81	4.87	12.69	3.75	17.81	4.76	15.31	3.74	<0.05 *

Abbreviations: * significant, IPQ-DIF: difficulty identifying feelings, DDF: difficulty describing feelings, TL a/c: timeline acute/chronic, TL c: timeline cyclical, CON: consequences, PC: personal control, CC: cure control, COH: coherence, ER: emotional representation.

**Table 7:** Post hoc test of psychometric data between PPPD and ANX.

Questionnaire	PPPD				ANX				*p*-Value
	T1		T2		T1		T2		
	M	SD	M	SD	M	SD	M	SD	
STAI-S	47.55	11.29	45.27	8.51	47.06	11.20	37.94	10.21	1.00
STAI-T	49.00	3.13	49.91	1.45	50.19	3.90	49.75	2.79	1.00
ACQ	1.74	0.51	1.44	0.24	1.92	0.72	1.56	0.41	0.82
BCQ	2.38	0.70	1.98	0.53	2.36	0.84	1.80	0.68	1.00
MI-A	1.69	0.78	1.36	0.42	2.31	0.96	1.87	1.00	0.12
MI-B	1.46	0.63	1.19	0.28	1.90	0.73	1.38	0.56	0.22
VSS-AA	25.91	10.52	24.64	10.92	24.06	11.42	18.19	6.78	0.70
VSS-VER	27.64	17.40	25.73	18.80	13.00	12.16	7.75	8.33	0.07
BDI-II	14.00	7.18	11.09	5.38	13.13	10.31	8.00	7.46	0.86
TAS-total	59.82	9.14	59.27	9.81	63.25	9.67	62.25	7.65	1.00
TAS-DIF	20.55	5.92	20.00	6.80	21.63	6.62	20.25	5.29	1.00
TAS-DDF	13.09	3.75	13.00	4.29	15.00	3.93	15.12	4.00	0.85
IPQ-TL a/c	16.73	3.95	14.73	2.90	16.25	2.41	15.44	3.98	1.00
IPQ-TL c	13.00	2.72	12.64	2.25	11.94	2.67	11.81	2.14	1.00
IPQ-CON	16.73	2.97	15.09	3.59	16.87	3.40	14.63	4.90	1.00
IPQ-PC	12.82	2.71	15.18	4.29	12.69	2.85	15.50	1.97	1.00
IPQ-CC	13.73	1.74	14.45	3.91	13.69	1.70	15.00	1.79	1.00
IPQ-COH	15.18	5.31	16.55	4.87	13.69	3.91	17.94	3.97	1.00
IPQ-ER	18.18	5.72	16.36	4.43	17.81	4.76	15.31	3.74	1.00

Abbreviations: IPQ-DIF: difficulty identifying feelings, DDF: difficulty describing feelings, TL a/c: timeline acute/chronic, TL c: timeline cyclical, CON: consequences, PC: personal control, CC: cure control, COH: coherence, ER: emotional representation.

**Table 8:** ANOVA with bootstrapping PPPD vs. ANX.

Questionnaire	PPPD		ANX		*p*-Value
	T2		T2		
	M	SD	M	SD	
VSS-VER	25.73	18.80	7.75	8.33	0.02 *

Abbreviations: M: mean, SD: standard deviation, * significant.

**Table 9:** ANOVA with bootstrapping PPPD with anxiety disorder vs. PPPD without anxiety disorder.

Questionnaire	PPPD		PPPD		*p*-Value
	Without AD (N = 8)		With AD (N = 6)		
	M	SD	M	SD	
BSQ (T1)	2.05	0.59	2.81	0.62	0.04 *

Abbreviations: M: mean, SD: standard deviation, * significant, AD: anxiety disorder.

**Table 10:** Wilcoxon Test T1 vs. T2 and effect sizes.

	KC-P	KC-A	PPPD		ANX	
Questionnaire	*p*-Value	*p*-Value	*p*-Value	Effect Size r	*p*-Value	Effect Size r
STAI-S	0.57	0.14	0.44	−0.07	<0.00 *	−0.22
STAI-T	0.91	0.33	0.24	−0.25	0.61	−0.09
ACQ	0.67	<0.01 *	0.31	−0.22	<0.05 *	−0.35
BCQ	0.31	0.02 *	0.24	−0.25	0.01 *	−0.49
MI-A	0.78	0.03 *	0.10	−0.35	0.20	−0.23
MI-B	0.66	0.12	0.31	−0.22	0.08	−0.31
VSS-AA	0.12	0.61	0.26	−0.24	0.03 *	−0.47
VSS-VER	0.08	0.65	0.18	−0.28	0.01 *	−0.38
VHQ	0.53	-	0.03 *	−0.46	-	-
BDI-II	0.83	0.02 *	0.22	−0.26	0.04 *	−0.37
TAS-overall	1.00	0.71	0.65	−0.10	0.53	−0.11
TAS-DIF	0.16	0.36	0.48	−0.15	0.30	−0.18
TAS-DDF	0.78	0.87	1.00	0.00	0.69	−0.07 *
IPQ-TL a/c	0.40	0.92	0.14	−0.32	0.48	−0.12
IPQ-TL c	0.85	0.62	0.86	−0.04	0.72	−0.06
IPQ-CON	0.68	0.77	0.18	−0.29	0.12	−0.27
IPQ-PC	0.89	0.84	0.04 *	−0.43	<0.01 *	−0.52
IPQ-CC	0.42	0.95	0.32	−0.21	0.02 *	−0.42
IPQ-COH	0.35	0.76	0.50	−0.14	<0.01 *	−0.53
IPQ-ER	0.50	0.76	0.10	−0.34	0.03 *	−0.38

Abbreviations: r: Pearson’s correlation coefficient, * significant, DIF: difficulty identifying feelings, DDF: difficulty describing feelings, TL a/c: timeline acute/chronic, TL c: timeline cyclical, CON: consequences, PC: personal control, CC: cure control, COH: coherence, ER: emotional representation. No effect-sizes were computed for the HC groups, because they didn’t receive any intervention.

**Table 11:** ROI Analysis Wilcoxon Test T1 vs. T2.

		ANX (N = 8)			PPPD (N = 9)			HC (N = 20)		
ROI		M	SD	*p*-Value	M	SD	*p*-Value	M	SD	*p*-Value
**AMY+**	T1	876.00	689.14	0.06 *	796.00	627.90	0.52	540.00	490.80	0.65
	T2	468.36	585.75		648.00	615.18		773.52	1778.28	
**HIP+**	T1	1691.46	1552.80	0.02 **	1415.50	1525.01	0.52	1020.13	951.51	0.20
	T2	1039.36	1374.97		955.64	836.17		613.78	737.50	
**THA+**	T1	5302.92	3651.63	0.01 **	4506.50	3499.92	0.77	3672.30	3957.79	0.60
	T2	3278.45	3133.10		3536.91	2918.98		2710.96	3232.71	
**CG+**	T1	5023.08	4328.03	0.33	4723.00	5731.77	0.68	4445.91	5743.18	0.74
	T2	3864.18	3712.94		5459.00	4990.92		2703.68	3590.26	
**IFG+**	T1	7136.50	4620.76	0.03 **	8518.00	5122.95	0.37	4797.17	3623.65	0.30
	T2	4457.36	3359.08		7732.00	4829.74		4057.95	4143.26	
**INS+**	T1	3005.50	2516.00	0.04 **	3775.90	3940.90	0.86	2541.17	3639.86	0.90
	T2	1473.27	1147.92		4273.36	4222.17		1444.18	1882.31	
**SMG+**	T1	6858.75	4937.74	0.16	8553.20	6467.69	0.77	6692.17	5124.60	0.68
	T2	5115.18	3313.07		9803.82	5868.81		4996.69	4668.07	

Abbreviations: ** significant, * trend-level. Groups: ANX = anxiety, PPPD = dizziness, HC = healthy controls. M = mean and SD = standard deviation, number of activated voxels in selected brain regions: AMY = amygdala, HIP = hippocampus, THA = thalamus, CG = cingulate gyrus, IFG = inferior gyrus, INS = Insula, SMG = supramarginal gyrus.

**Table 12:** MANOVA with bootstrapping: ANX group, ROI—Amygdala—pre-/post-intervention.

Questionnaire	ANX				*p*-Value
	Pre (N = 9)		Post (N = 9)		
	M	SD	M	SD	
AMY+	322.22	450.33	1035.78	715.36	0.006 *

Abbreviations: M: mean, SD: standard deviation, * significant.

**Table 13:** MANOVA with bootstrapping: PPPD group, ROI—Hippocampus—pre-/post-intervention.

Questionnaire	PPPD				*p*-Value
	Pre (N = 9)		Post (N = 9)		
	M	SD	M	SD	
HIP+	1415.50	1525.01	955.64	836.17	0.009 *

Abbreviations: M: mean, SD: standard deviation, * significant.

## Data Availability

The datasets generated for this study are available on request to the corresponding author.

## Supplementary Materials

The following supporting information can be downloaded at: https://www.mdpi.com/article/10.3390/diagnostics15141729/s1, Table S1. PPPD—T1 vs. T2; Table S2. ANX—T1 vs. T2; Table S3. HC—T1 vs. T2; Table S4. PPPD vs. ANX—T2; Table S5. PPPD vs. HC-P—T2; Table S6. ANX vs. HC-A—T2.
