# Supplementary material for: Diagnostics and Group Therapy in Patients with Persistent Postural-Perceptual Dizziness and Anxiety Disorder: Biomarkers and Neurofunctional Correlates of Underlying Treatment Effects
Source: Diagnostics (Basel). 2025 Jul 8;15(14):1729. doi: 10.3390/diagnostics15141729 (PMC12293719; doi:10.3390/diagnostics15141729)
Supplement: Supplementary file 1 [file diagnostics-15-01729-s001.zip › diagnostics-3606096-supplementary.pdf]

**Table S1.** *PPPD - T1 vs. T2*, neuronal responses during the emotion-associated task (negative emotional-associated pictures minus neutral pictures; clusters of >30 voxels, q(FDR) < 0.05, T-score: 8 to -8).

| | | | **Centre of gravity** | | | **Size** | **t-score** | |
|---|---|---|---|---|---|---|---|---|
| **Brain region** | **side** | **BA** | **x** | **y** | **z** | | **Ø** | **max** |
| **decrease of neuronal activation (T1>T2)** | | | | | | | | |
| Precentral Gyrus | R | 4 | 28.13 | -27.39 | 52.25 | 11166 | 3.48 | 5.11 |
| Postcentral Gyrus | | 3 | | | | | | |
| Medial Frontal Gyrus | L | 9 | -4.24 | 46.59 | 31.78 | 1439 | 3.25 | 4.04 |
| Precentral Gyrus | L | 9 | -38.32 | -5.95 | 48.4 | 7476 | 3.46 | 5.68 |
| Middle Frontal Gyrus | | 6 | | | | | | |
| Cuneus | L | 17/18 | -4.29 | -88.36 | 10.89 | 17562 | 3.48 | 5.24 |
| Hippocampus | L | * | -28.26 | -36.99 | 6.53 | 820 | 3.52 | 4.83 |
| Parahippocampal Gyrus | R | 30 | 18.96 | -42.58 | -1.02 | 1094 | 3.20 | 3.98 |
| Superior Temporal Gyrus | R | 22 | 52.92 | -29.87 | 0.88 | 2946 | 3.45 | 4.52 |
| Middle Temporal Gyrus/ Superior Temporal Gyrus | R | 37 | 47.16 | -63.87 | 4.11 | 1041 | 3.52 | 4.86 |
| | L | 21/22 | -58.98 | -32.81 | 0.73 | 2003 | 3.36 | 4.21 |
| Declive | L/R | * | 0.76 | -68.99 | -24.72 | 18770 | 3.7 | 6.50 |
| Uvula | L/R | | | | | | | |
| Tuber | L | | | | | | | |
| Pyramis | L/R | | | | | | | |
| **increase of neuronal activation (T1<T2)** | | | | | | | | |
| Inferior Parietal Lobule/ Supramarginal Gyrus | R | 40 | 55.3 | -38.76 | 35.15 | 3183 | -3.63 | -5.03 |
| | L | 40 | -61.17 | -29.8 | 24.1 | 840 | -3.17 | -3.61 |
| Middle Temporal Gyrus | R | 39 | 46.24 | -65.57 | 26.93 | 1134 | -3.53 | -4.93 |
| Posterior Cingulate Cortex | R | 23 | -3.61 | -3.61 | -3.61 | 949 | -3.33 | -4.24 |

Abbreviations: BA: Brodmann area; side: hemisphere; L: left; R: right; max: maximal t-score; Ø: average t-score; size: cluster size; voxels: number of activated voxels; x: Talairach coordinate x-axis; y: Talairach coordinate y-axis; z: Talairach coordinate z-axis.

**Table S2.** ANX - T1 vs. T2, neuronal responses during the emotion-associated task (negative emotional-associated pictures minus neutral pictures; clusters of >30 voxels, q(FDR) < 0.05, T-score: 8 to -8).

| | | | Centre of gravity | | | Size | t-score | |
|---|---|---|---|---|---|---|---|---|
| **Brain region** | **side** | **BA** | **x** | **y** | **z** | | **Ø** | **max** |
| **decrease of neuronal activation (T1>T2)** | | | | | | | | |
| Precentral Gyrus | R | 6 | 43.57 | 2.33 | 35.69 | 4273 | 3.49 | 5.89 |
| | R | | 37.57 | -10.29 | 55.28 | 857 | 2.85 | 3.58 |
| | L | | -40.68 | 1.48 | 36.11 | 6886 | 3.36 | 5.52 |
| Inferior Frontal Gyrus | R | 45 | 44.22 | 24.89 | 16.48 | 6887 | 3.57 | 6.55 |
| | R | 47 | 43.77 | 18.8 | 0.77 | 4859 | 3.59 | 6.70 |
| | R | | 39.77 | 13.66 | -21.3 | 3547 | 3.48 | 5.66 |
| | L | | -38.18 | 24.62 | 2.01 | 3950 | 3.09 | 5.14 |
| Superior Frontal Gyrus | L | 6 | 0.24 | 9.56 | 58.49 | 2879 | 2.88 | 3.92 |
| Medial Frontal Gyrus | L/R | 10 | -0.05 | 56.84 | 1.86 | 1250 | 3.01 | 4.03 |
| | | | | | | | | |
| Precuneus | L | 7 | -17.39 | -71.99 | 42.23 | 14402 | 4.11 | 7.12 |
| | R | | 21.15 | -66.11 | 43.74 | 23486 | 3.75 | 7.47 |
| Inferior Parietal Lobule | L | 40 | -32.11 | -47.46 | 51.55 | 3262 | 3.09 | 4.26 |
| Gyrus Lingualis | R | 18/17 | 22.81 | -81.81 | -0.78 | 31688 | 5.18 | 9.72 |
| | L | 17 | -16.95 | -86.85 | 2.84 | 48362 | 5.01 | 9.51 |
| | L | 19 | -15.21 | -63.97 | -3.61 | 27820 | 3.93 | 7.37 |
| Middle Occipital Gyrus | R | 18 | 39.86 | -83.77 | 1.36 | 4327 | 6.58 | 10.16 |
| Superior Temporal Gyrus | R | 38 | 39.77 | 13.66 | -21.3 | 3547 | 3.48 | 5.66 |
| Middle Temporal Gyrus | R | 21 | 49.67 | -6.74 | -13.9 | 3998 | 3.31 | 5.56 |
| | R | 37 | 46.31 | -57.38 | 0.52 | 34880 | 5.67 | 11.82 |
| | L | | -42.52 | -61.55 | -1.38 | 34724 | 5.30 | 11.70 |
| Parahippocampal Gyrus | R | 36 | 36.23 | -32.08 | -13.61 | 1479 | 3.95 | 7.07 |
| | R | 19 | 21.37 | -54.53 | -2.33 | 19294 | 4.39 | 9.36 |
| | L | 36 | -25.33 | -33.71 | -8.29 | 8289 | 3.48 | 6.43 |
| | L | AMY | -20.17 | -6.53 | -8.31 | 6799 | 3.48 | 6.35 |
| | L | HIP | -35.78 | -12.34 | -21.48 | 517 | 2.80 | 3.61 |
| Nucleus Lentiformis | R | PUT | 21.47 | -0.8 | -8.05 | 4919 | 3.16 | 5.13 |
| | L | LGP | -20.17 | -6.53 | -8.31 | 6799 | 3.48 | 6.35 |
| Insula | L | 13 | -38.18 | 24.62 | 2.01 | 3950 | 3.09 | 5.14 |
| Thalamus | R | * | 19.61 | -28.36 | -4.25 | 5221 | 3.17 | 4.88 |

| | | | | | | | | |
|---|---|---|---|---|---|---|---|---|
| **increase of neuronal activation (T1<T2)** | | | | | | | | |
| Middle Frontal Gyrus | R | 8 | 36.51 | 27.56 | 44.67 | 2632 | -3.00 | -4.65 |
| | L | | -42.89 | 24.25 | 37.35 | 1076 | -3.03 | -4.33 |
| Medial Frontal Gyrus | R | 9 | 24.53 | 41.79 | 21.78 | 2238 | -2.98 | -4.30 |
| Superior Frontal Gyrus | L | 9 | -30.29 | 48.67 | 27.81 | 1664 | -3.04 | -4.63 |
| Precentral Gyrus | L | 6 | -59.26 | -3.69 | 23.96 | 2483 | -3.05 | -4.24 |
| Inferior Parietal Lobule | R | 39 | 42.28 | -65.67 | 40.25 | 1086 | -2.96 | -3.88 |
| Precuneus | L | 19 | -11.55 | -54.3 | 28.44 | 1985 | -3.31 | -5.24 |
| Posterior Cingulate Cortex | R | 23 | 3.92 | -29.56 | 24.91 | 3251 | -2.79 | -3.51 |

Abbreviations: BA: Brodmann area; side: hemisphere; L: left; R: right; max: maximal t-score; Ø: average t-score; size: cluster size; voxels: number of activated voxels; x: Talairach coordinate x-axis; y: Talairach coordinate y-axis; z: Talairach coordinate z-axis), AMY=Amygdala, HIP=Hippocampus, PUT=Putamen, LGP=Lateral Globus Pallidus.

**Table S3.** *HC - T1 vs. T2*, neuronal responses during the emotion-associated task (negative emotional-associated pictures minus neutral pictures; clusters of >30 voxels, q(FDR) < 0.05, T-score: 8 to -8).

| | | | **Centre of gravity** | | | **Size** | **t-score** | |
|---|---|---|---|---|---|---|---|---|
| **Brain region** | **side** | **BA** | **x** | **y** | **z** | | **Ø** | **max** |
| **decrease of neuronal activation (T1<T2)** | | | | | | | | |
| Superior Frontal Gyrus | L | 8 | -19.31 | 18.5 | 48.61 | 745 | 2.90 | 4.05 |
| | R | 10 | 24.26 | 48.26 | 21.24 | 5184 | 3.12 | 5.89 |
| Inferior Frontal Gyrus | L | 47 | -43.01 | 18.12 | 1.55 | 6258 | 3.43 | 6.30 |
| | L | | -32.11 | 11.29 | -10.72 | 483 | 2.77 | 4.55 |
| | R | 45 | 44.79 | 22.05 | 13.73 | 13108 | 3.43 | 6.84 |
| | R | 9 | 43.86 | 16.49 | 24.45 | 14468 | 3.59 | 6.90 |
| Medial Frontal Gyrus | L | 9 | -1.93 | 48.61 | 48.61 | 22035 | 4.04 | 7.34 |
| | R | 6 | 19.09 | 5.58 | 53.64 | 1069 | 2.59 | 3.16 |
| Middle Frontal Gyrus | L | 9 | -39.13 | 14.99 | 24.75 | 5458 | 3.30 | 6.46 |
| | L | 6 | -37.54 | -3.62 | 45.69 | 2630 | 3.30 | 5.71 |
| Precentral Gyrus | R | 6 | 36.61 | 1.34 | 39.38 | 6442 | 3.46 | 6.90 |
| Precuneus | L | 7 | -12.26 | -50.92 | 50.05 | 15175 | 3.29 | 6.63 |
| | R | | 17.5 | -54.71 | 48.91 | 17542 | 4.03 | 7.42 |
| Inferior Parietal Lobule/ Supramarginal Gyrus | R | 40 | 57.71 | -31.15 | 33.57 | 1540 | 2.90 | 4.05 |
| Cuneus | R/L | 18 | -0.98 | -91.49 | 5.59 | 44211 | 8.60 | 15.53 |
| | R | 19 | 18.69 | -78.47 | 32.48 | 8612 | 6.07 | 10.99 |
| | L | | -16.62 | -77.68 | 34.67 | 14864 | 5.03 | 8.62 |
| Lingual Gyrus | L | 18 | -14.46 | -70.0 | 4.0 | 34291 | 5.78 | 11.31 |
| | R | | 16.38 | -69.63 | 3.41 | 35551 | 6.48 | 14.90 |
| Middle Occipital Gyrus | R | 37 | 42.93 | -67.46 | 2.68 | 25581 | 10.90 | 18.99 |

| | | | | | | | | |
|---|---|---|---|---|---|---|---|---|
| Middle Temporal Gyrus | L | 37 | -42.85 | -67.91 | 3.37 | 29159 | 8.10 | 15.36 |
| Superior Temporal Gyrus | L | 22 | -49.47 | -44.8 | 11.21 | 10863 | 4.12 | 7.30 |
| | R | | 51.39 | -42.28 | 11.4 | 24978 | 5.52 | 16.53 |
| | R | 38 | 46.92 | 3.56 | -9.32 | 4888 | 3.22 | 5.63 |
| Fusiform Gyrus | L | 37 | -28.55 | -41.46 | -11.93 | 22396 | 4.88 | 13.60 |
| Insula | L | 13 | -41.29 | -14.27 | -7.64 | 4180 | 3.00 | 5.14 |
| | L | | -28.73 | 24.29 | 4.11 | 728 | 4.08 | 3.02 |
| Parahippocampal Gyrus | R | 37 | 27.34 | -43.99 | -11.89 | 21647 | 5.61 | 14.52 |
| | R | HIP | 28.81 | -18.23 | -8.0 | 13419 | 3.52 | 6.88 |
| Lentiform Nucleus | L | LGP | -22.67 | -8.96 | -7.77 | 5424 | 3.12 | 6.26 |
| | R | PUT | 17.33 | 4.76 | -0.18 | 10289 | 3.14 | 5.07 |
| | L | PUT | -25.98 | -0.46 | 5.77 | 772 | 2.68 | 3.59 |
| Cingulate Gyrus | L | 32 | -0.49 | 20.91 | 39.27 | 21867 | 5.75 | 3.15 |
| | R | 24 | 2.22 | -12.54 | 38.43 | 1760 | 2.87 | 4.28 |
| Anterior Cingulate Gyrus | L | 32 | -3.15 | 48.59 | 1.85 | 7263 | 5.95 | 3.71 |
| Posterior Cingulate Gyrus | L | 29 | -7.46 | -48.3 | 14.32 | 4920 | 3.36 | 6.49 |
| | R | | 11.68 | -47.56 | 9.5 | 7180 | 3.87 | 6.52 |
| Nucleus caudatus | L | Head | -6.85 | 1.81 | 4.9 | 4560 | 28 | 3.00 |
| Thalamus | L | PUL | -12.58 | -30.14 | 2.83 | 1790 | 3.53 | 5.35 |
| | L | MDN | -8.15 | -14.25 | 10.9 | 1925 | 2.89 | 3.96 |
| | R | MDN | 6.95 | -9.67 | 12.36 | 1636 | 3.08 | 4.52 |
| | R | PUL | 22.44 | -23.37 | 10.01 | 1318 | 2.80 | 4.24 |
| Cerebellar Tonsil | R | * | 24.97 | -39.75 | -31.7 | 3506 | 3.78 | 6.90 |
| | L | | -2.06 | -52.84 | -30.63 | 4000 | 3.89 | 7.62 |
| | L | | -20.25 | -36.54 | -36.1 | 926 | 2.90 | 3.85 |
| Declive | R | * | 19.44 | -66.78 | -24.37 | 15230 | 4.96 | 10.94 |
| | L | | -19.37 | -75.81 | -21.08 | 18942 | 6.69 | 14.15 |
| Pons | R | * | 4.27 | -29.94 | -34.11 | 1669 | 2.67 | 3.59 |
| | L | * | -6.34 | -28.3 | -22.79 | 667 | 4.36 | 3.04 |

**increase of neuronal activation (T1<T2)**

| | | | | | | | | |
|---|---|---|---|---|---|---|---|---|
| Inferior Frontal Gyrus | R | 44 | 60.32 | 7.05 | 20.54 | 1594 | -2.91 | -4.22 |
| Middle Frontal Gyrus | L | 8/9 | -37.2 | 31.04 | 37.32 | 1077 | -3.88 | -2.90 |
| Inferior Parietal Lobule | R | 40 | 44.58 | -52.87 | 46.54 | 4451 | -3.35 | -5.54 |
| | L | | -44.5 | -53.43 | 43.87 | 2821 | -2.99 | -4.67 |
| Postcentral Gyrus | L | 3 | -45.07 | -18.97 | 48.83 | 6445 | -3.01 | -5.10 |
| Insula | L | 13 | -41.01 | -8.4 | 18.28 | 921 | -2.70 | -3.44 |

Abbreviations: BA: Brodmann area; side: hemisphere; L: left; R: right; max: maximal t-score; Ø: average t-score; size: cluster size; voxels: number of activated voxels; x: Talairach coordinate x-axis; y: Talairach coordinate y-axis; z: Talairach coordinate z-axis), HIP=Hippocampus, LGP=Lateral Globus Pallidus, MDN=Medial Dorsal Nucleus, PUL= Pulvinar, PUT=Putamen.

**Table S4.** *PPPD vs. ANX - T2*, neuronal responses during the emotion-associated task (negative emotional-associated pictures minus neutral pictures; clusters of >30 voxels, q(FDR) < 0.01, T-score: 3,48 to 8).

| | | | Centre of gravity | | | Size | t-score | |
|---|---|---|---|---|---|---|---|---|
| **Brain region** | **side** | **BA** | **x** | **y** | **z** | | **Ø** | **max** |
| **PPPD > ANX (T2)** | | | | | | | | |
| Middle Frontal Gyrus | L | 6 | -27.47 | -8.67 | 56.15 | 1640 | 4.16 | 5.51 |
| Inferior Frontal Gyrus | L | 9 | -46.39 | 7.33 | 28.03 | 2215 | 4.73 | 8.45 |
| Precentral Gyrus | R | 9 | 43.63 | 7.7 | 37.17 | 3615 | 4.32 | 6.12 |
| Inferior Parietal Lobule | L | 40 | -57.64 | -36.52 | 29.13 | 2068 | 4.16 | 6.27 |
| Supramarginal Gyrus | R | 40 | 56.29 | -39.57 | 31.19 | 1523 | 4.07 | 5.15 |
| Precuneus | L | 7 | -5.42 | -64.75 | 54.42 | 830 | 3.89 | 4.66 |
| | L | | -23.77 | -66.57 | 41.65 | 6864 | 4.02 | 5.58 |
| Precuneus/Cuneus | R | 31/18 | 30.22 | -73.92 | 22.23 | 5404 | 4.14 | 5.97 |
| Fusiform Gyrus | L | 19 | -36.61 | -71.64 | -7.93 | 7721 | 4.51 | 8.59 |
| | L | 37 | -40.45 | -49.31 | -15.35 | 546 | 3.86 | 4.68 |
| Superior Temporal Gyrus | R | 41 | 52.24 | -21.3 | 11.82 | 1123 | 3.91 | 4.83 |
| Inferior Temporal Gyrus | R | 37 | 39.65 | -60.07 | -5.26 | 14533 | 4.67 | 9.89 |
| Parahippocampal Gyrus | L | AMY | -28.37 | -7.41 | -16.36 | 1107 | 3.73 | 4.35 |
| Culmen | L | * | -40.03 | -45.16 | -28.01 | 334 | 4.16 | 5.09 |
| **ANX > PPPD (T2)** | | | | | | | | |
| - | - | - | - | - | - | - | - | - |

Abbreviations: BA: Brodmann area; side: hemisphere; L: left; R: right; max: maximal t-score; Ø: average t-score; size: cluster size; voxels: number of activated voxels; x: Talairach coordinate x-axis; y: Talairach coordinate y-axis; z: Talairach coordinate z-axis).

**Table S5.** *PPPD vs. HC-P - T2*, neuronal responses during the emotion-associated task (negative emotional-associated pictures minus neutral pictures; clusters of >30 voxels, q(FDR) < 0.01, T-score: 8 to -8).

#

| | | | Centre of gravity | | | Size | t-score | |
|---|---|---|---|---|---|---|---|---|
| **Brain region** | **side** | **BA** | **x** | **y** | **z** | | **Ø** | **max** |
| **PPPD > HC-P (T2)** | | | | | | | | |
| Superior Frontal Gyrus | R | 9 | 26.18 | 46.78 | 27.05 | 15207 | 4.59 | 8.30 |
| Middle Frontal Gyrus | R | 6 | 21.55 | -1.53 | 61.77 | 3072 | 4.29 | 6.21 |
| | L | | -26.26 | -3.01 | 60.2 | 3582 | 3.82 | 5.78 |
| | L | 9 | -36.35 | 27.46 | 36.91 | 1465 | 3.61 | 4.71 |
| | L | 10 | -35.33 | 41.53 | 11.92 | 6183 | 3.56 | 4.58 |

| | | | | | | | | |
|---|---|---|---|---|---|---|---|---|
| Medial Frontal Gyrus | L | | -11.95 | 49.85 | 6.38 | 1995 | 3.63 | 5.32 |
| Inferior Frontal Gyrus | L | 9 | -45.59 | 8.69 | 8.69 | 1837 | 3.78 | 5.93 |
| Precentral Gyrus | R | | 41.08 | 9.76 | 38.53 | 8934 | 4.79 | 9.01 |
| | | | | | | | | |
| Precuneus | R | 7 | 8.28 | -63.47 | 54.92 | 4332 | 3.84 | 5.67 |
| | R | 31 | 14.46 | -68.28 | 27.55 | 5779 | 4.25 | 6.40 |
| | L | 7 | -10.66 | -73.35 | 36.12 | 10379 | 4.54 | 8.38 |
| Superior Parietal Lobule | L | 7 | -31.69 | -52.6 | 52.93 | 8022 | 3.91 | 6.16 |
| | L | 40 | -52.83 | -41.26 | 28.1 | 14068 | 4.27 | 7.09 |
| Supramarginal Gyrus<br>Superior Temporal Gyrus | R | 40 | 50.3 | -44.77 | 30.62 | 17708 | 4.72 | 7.78 |
| Middle Temporal Gyrus | R | 37 | 48.3 | -54.84 | 0.86 | 20795 | 5.00 | 10.25 |
| Fusiform Gyrus | L | 37 | -40.06 | -51.8 | -12.08 | 2665 | 3.72 | 5.35 |
| | R | 19 | 26.92 | -80.73 | -14.24 | 2950 | 4.28 | 8.03 |
| | R | | 26.42 | -80.71 | -14.4 | 2996 | 4.27 | 8.03 |
| Middle Occipital Gyrus | L | 19 | -45.22 | -71.14 | 3.12 | 3501 | 4.11 | 6.85 |
| Lingual Gyrus | R | 18 | 27.52 | -69.19 | -5.07 | 853 | 4.29 | 7.22 |
| Nucleus lentiformis<br>Claustrum<br>Amygdala | R | PUT | 31.17 | 4.78 | -4.76 | 32306 | 4.15 | 7.56 |
| Thalamus | L | VLN | -7.46 | -8.95 | 5.37 | 1739 | 3.40 | 4.32 |
| Cingulate Gyrus | L | 31 | -12.52 | -37.7 | 39.74 | 1317 | 4.20 | 6.46 |
| | R | | 8.19 | -40.46 | 35.51 | 1758 | 3.68 | 5.05 |
| Posterior Cingulate Gyrus | L | 30 | -8.73 | -61.42 | 12.02 | 1149 | 3.47 | 4.52 |
| Insula | L | 13 | -41.82 | 11.45 | 2.62 | 7415 | 4.07 | 5.79 |
| | L | | -40.88 | -14.09 | -0.86 | 1101 | 3.56 | 4.98 |
| Culmen | L | * | -18.7 | -47.92 | -3.62 | 845 | 3.60 | 4.65 |

**HC-P>PPPD (T2)**

| | | | | | | | | |
|---|---|---|---|---|---|---|---|---|
| - | - | - | - | - | - | - | - | - |

Abbreviations: BA: Brodmann area; side: hemisphere; L: left; R: right; max: maximal t-score; Ø: average t-score; size: cluster size; voxels: number of activated voxels; x: Talairach coordinate x-axis; y: Talairach coordinate y-axis; z: Talairach coordinate z-axis), PUT=Putamen, VLN=Ventral Lateral Nucleus.

**Table S6.** ANX *vs. HC-A - T2*, neuronal responses during the emotion-associated task (negative emotional-associated pictures minus neutral pictures; clusters of >30 voxels, q(FDR) < 0.01, T-score: 8 to -8).

| | | | Centre of gravity | | | Size | t-score | |
|---|---|---|---|---|---|---|---|---|
| **Brain region** | **side** | **BA** | **x** | **y** | **z** | | **Ø** | **max** |
| **ANX > HC-A (T2)** | | | | | | | | |
| Middle Frontal Gyrus | R | 9 | 31.88 | 38.59 | 26.68 | 4844 | 3.77 | 5.17 |

| | | | | | | | | |
|---|---|---|---|---|---|---|---|---|
| | L | 10 | -35.59 | 47.7 | 17.17 | 3816 | 3.92 | 5.33 |
| Medial Frontal Gyrus | L | 32 | 0.01 | 30.65 | 30.1 | 21759 | 4.17 | 7.49 |
| Cingulate Gyrus | L | 31 | -1.38 | -36.53 | 33.46 | 10583 | 3.85 | 5.36 |
| Inferior Frontal Gyrus | R | 44 | 47.79 | 13.14 | 4.68 | 2690 | 3.93 | 5.49 |
| | L | | -48.15 | 12.8 | 3.58 | 4605 | 4.11 | 5.89 |
| Cuneus | R | 18 | 6.15 | -75.5 | 28.54 | 3165 | 4.04 | 6.06 |
| Insula | R | 13 | 43.07 | -13.93 | -1.48 | 1327 | 4.08 | 5.65 |
| | L | | -37.02 | -18.12 | 10.02 | 2422 | 3.83 | 4.98 |
| | L | | -48.15 | 12.8 | 3.58 | 4605 | 3.92 | 5.33 |

**HC-A > ANX (T2)**

| | | | | | | | | |
|---|---|---|---|---|---|---|---|---|
| Precentral Gyrus | R | 6 | 44.02 | 5.89 | 32.03 | 1206 | 3.70 | 4.73 |
| | L | | -48.8 | 1.76 | 36.61 | 1443 | 4.40 | 6.66 |
| Superior Parietal Lobule | L | 7 | -26.11 | -53.87 | 53.08 | 4533 | 4.18 | 5.93 |
| | R | | 27.73 | -57.89 | 45.61 | 11518 | 4.32 | 6.98 |
| Inferior Temporal Gyrus | L | 37 | -44.8 | -64.28 | -5.3 | 17515 | 4.89 | 9.18 |
| Middle Occipital Gyrus | R | 37 | 42.82 | -68.15 | 0.27 | 15295 | 4.60 | 9.28 |
| Gyrus fusiformis | R | 37 | 35.08 | -61.89 | -17.53 | 3483 | 4.28 | 6.22 |

Abbreviations: BA: Brodmann area; side: hemisphere; L: left; R: right; max: maximal t-score; Ø: average t-score; size: cluster size; voxels: number of activated voxels; x: Talairach coordinate x-axis; y: Talairach coordinate y-axis; z: Talairach coordinate z-axis).
